# Laparoscopic Removal of a Non-Adjustable Gastric Band and Conversion to a Gastric Sleeve: A Case Report

**DOI:** 10.7759/cureus.62064

**Published:** 2024-06-10

**Authors:** Ricardo Xavier Cuellar-Tamez, Milton Alberto Muñoz Leija, Cristian Santiago Ramírez-López, José Andrés Patiño-Gallegos, Omar F Wong Rodríguez

**Affiliations:** 1 Surgery Department, Hospital Zambrano Hellion TecSalud, San Pedro Garza García, MEX; 2 General Surgery Department, Universidad de Monterrey, San Nicolás de los Garza, MEX

**Keywords:** bariatric and metabolic surgery, bariatric revision, gastric sleeve surgery, bariatric surgery complications, laparoscopic gastric sleeve gastrectomy, non-adjustable band

## Abstract

Obesity has long been recognized as a global epidemic. One of the most effective treatments is bariatric surgery. Since the first modern procedure was reported, it has evolved over time, and multiple techniques have emerged. More than 20 years ago, one of the most widely used techniques was the non-adjustable gastric band (NAGB), which showed very promising short-term results. However, over time, it became apparent that it was not as effective in the long term. Associated gastrointestinal symptoms, such as reflux and constant vomiting, along with considerable weight regain, caused this technique to fall out of favor and be replaced by other procedures like the gastric sleeve (GS). Although the technique has fallen into disuse and is no longer recommended in the literature, there are still patients with associated complications. Few recent cases associated with these complications have been reported. Most undergo band removal, and whether to perform another procedure remains with limited evidence. We present the case of a patient who underwent an NAGB procedure 10 years ago and later experienced symptoms (reflux) and weight regain. She successfully underwent band removal and conversion to a GS at our institute in Mexico.

## Introduction

Obesity has been recognized as a global epidemic for more than 20 years, although records of procedures associated with obesity date back to medieval times [1]. Since Dr. Kremen's report of the first surgical procedure in 1954, bariatric surgery has demonstrated effectiveness and provided new insights into this disease [1,2]. Over time, new, safer, and more effective techniques were developed, especially with the introduction of laparoscopy. However, history has shown that not all cases have been complete successes.

One of these techniques was non-adjustable gastric banding (NAGB), introduced by Dr. Molina in the early 1980s, who performed this procedure on more than 7,000 patients [3]. The surgery involved performing gastric segmentation using a band of prosthetic material (polypropylene, Gore-Tex, Dacron, and silastic tubing) around the stomach, creating a weight loss mechanism through restriction [4,5]. No difference has been reported between the materials, as they all have the characteristics of being flexible and soft [6]. Initially, it showed very good results, such as significant short-term weight loss and very few perioperative complications. However, the associated gastrointestinal symptoms (oral intolerance, dysphagia, persistent nausea, vomiting, reflux, and long-term weight regain) made the procedure not recommended. Over time, the procedure fell into disuse due to the associated complications [5,7]. However, there are still patients who underwent this procedure years ago.

The objective of this study is to present the case of a patient from northeastern Mexico (in the city of Monterrey, Nuevo León) who came to our hospital for the evaluation and removal of an NAGB due to associated symptoms. The patient was successfully treated by NAGB removal and conversion to a gastric sleeve (GS). This case has been reported in line with the Surgical Case Report (SCARE) criteria [8].

## Case presentation

A 39-year-old female patient with no significant medical history, but with a surgical history of NAGB placement via laparotomy approximately 10 years ago in northeastern Mexico, presented to our clinic with reflux, dysphagia, and weight regain over the past four years. Her present BMI was 34.9. Preoperative studies were conducted, including an endoscopy that showed no evidence of erosion, along with preoperative preparation according to international and institutional protocols. A decision was made to perform a revision surgery and conversion to GS.

The procedure was performed under general anesthesia. The patient was placed in the French position with the surgeon standing in front of her, assisted by two assistants, one on the left and one on the right. Using the Veress technique, pneumoperitoneum was established at 15 mmHg at Palmer’s point. A 5 mm trocar was placed in the supraumbilical midline. Diagnostic laparoscopy was performed, and under direct vision, the remaining ports were placed: a 5 mm subxiphoid port, a 12 mm right hypochondrial port, a 12 mm left hypochondrial port, and a 5 mm left flank port.

**Figure 1:**
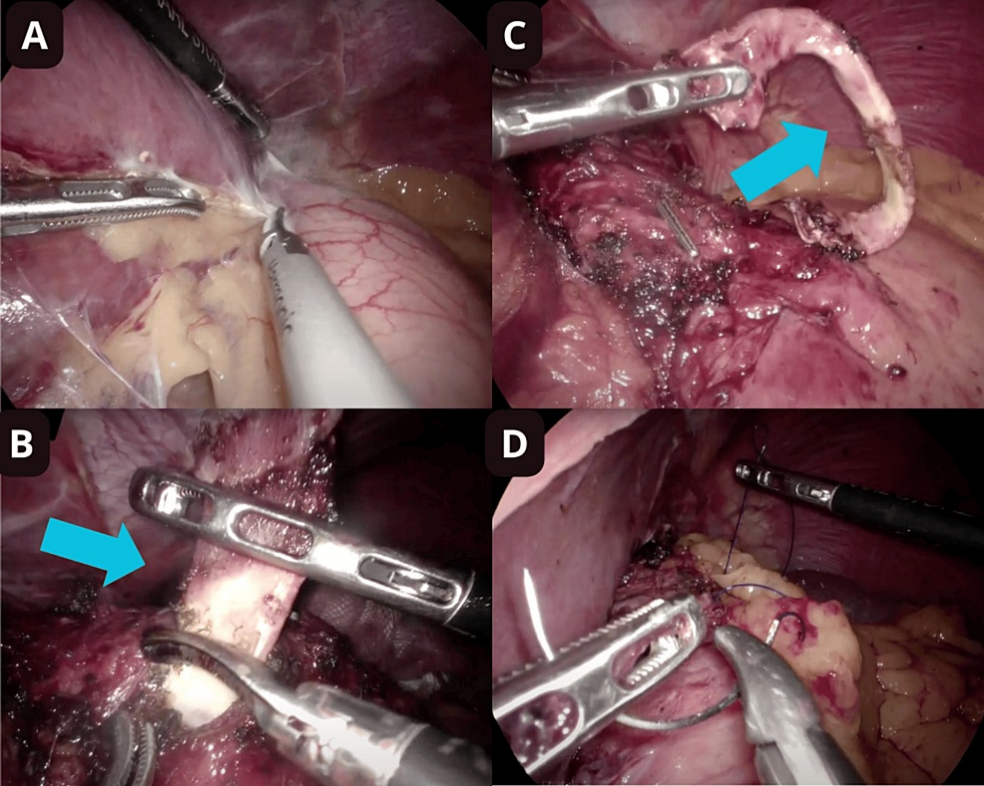
Laparoscopic view of the upper left quadrant in the abdomen. (A) Adhesions between the stomach and liver. (B) Stomach with erosion due to the non-adjustable band (blue arrow). (C) Non-adjustable band removed (blue arrow). (D) Reinforcement of the new stomach with an omentum patch using a non-absorbable suture.

Adhesions from the previous surgery and the NAGB were dissected using ultrasonic energy, ensuring hemostasis. Adhesions between the stomach and liver were released using blunt dissection and ultrasonic energy (Figure 1A). The non-adjustable gastric band was found adhered at the cardia (Figure 1B) and was removed using ultrasonic energy. Hemostasis was verified, and the band was removed (Figure 1C). A conventional GS was performed, severing the short gastric vessels along the greater curvature. A 36 Fr orogastric tube was inserted to calibrate the stomach. Stapling was performed using an Ethicon stapler (Johnson & Johnson, New Jersey, USA). Bleeding was checked, and the orogastric tube was removed to reinforce the staple line with a non-absorbable suture (Prolene 2-0). An omentum patch was placed on the upper third of the new stomach and secured with Prolene 2-0 (Figure 1D). The 12 mm ports were closed with non-absorbable sutures, and the skin was closed with monofilament.

**Figure 2:**
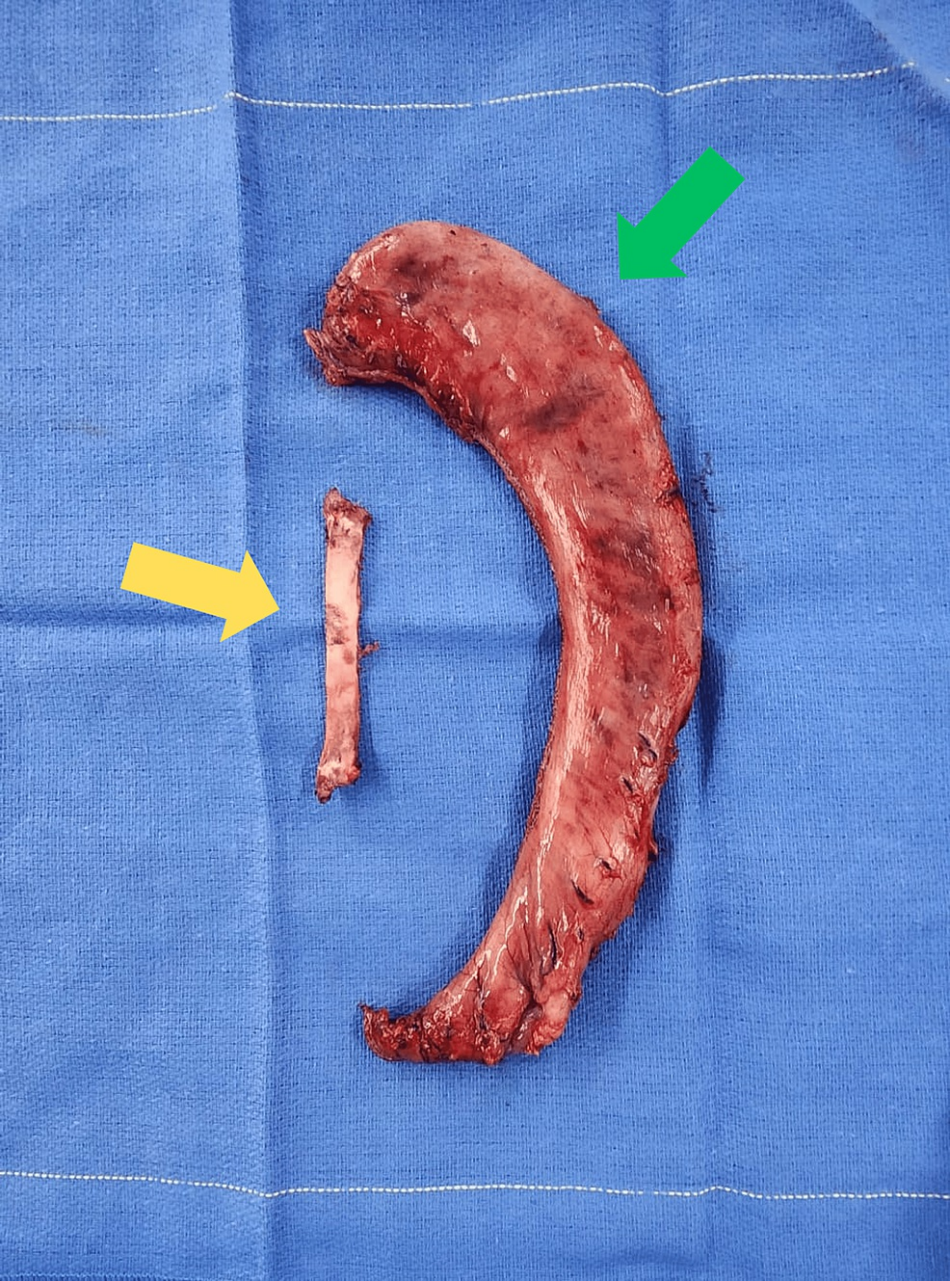
Non-adjustable band removed (yellow arrow) and stomach removed (green arrow).

The resected specimens, including the NAGB and removed stomach, were sent to pathology (Figure 2).

Within the first 24 postoperative hours, a barium swallow test was performed to assess the new stomach, which showed no leaks or herniation. 

On postoperative day two, the patient was discharged due to an adequate evolution. The patient was followed up for 24 months, during which she showed improvement in her quality of life and lost 70% of her excess weight.

## Discussion

The NAGB was a very popular procedure in the early 2000s in our region. A lot of the population with obesity had this option as a treatment. Moreover, a large number of procedures were performed [5,9]. Many of them were done conventionally. With the popularization of other more effective bariatric procedures and laparoscopic surgery, the NAGB fell into oblivion [5,6]. However, almost 20 years later, sporadic cases like that of our patient still occur.

In 2014, Balogh et al. [5] reported a series of cases of 11 patients in the USA who experienced complications due to NAGB. Over 70% of them had vomiting as a complication and weight regain. They also had solid and liquid intolerance and reflux. Endoscopic treatment was performed in two patients, band removal alone in four patients, and band removal with laparoscopic partial gastrectomy and Roux-en Y gastrojejunostomy (RYGB) in three patients. Only one patient underwent band removal and conversion to GS. This last case showed adequate weight loss in the first year. However, after two years, there was weight regain. However, this case did not have a favorable outcome, unlike our patient, who showed symptom improvement and adequate weight loss at 24 months.

One reason for this could be that, in recent years, there has been an increase in knowledge about obesity and the effects of bariatric surgery, owing to the emergence of new techniques and the large number of long-term follow-up studies published on patients. In addition, molecular and histological studies have focused on the impact of comorbidities. Another approach that has significantly changed in recent years is the multidisciplinary team approach for patients [10-12]. Previously, the focus was solely on the surgery itself. However, now preparatory evaluations are conducted, patient selection criteria are in place, and post-surgery follow-up includes nutritional and psychological support. Moreover, regular follow-up consultations with the multidisciplinary bariatric team collectively ensure proper patient weight loss [10]. The use of multidisciplinary teams in bariatric surgery is recommended [11,12]. Our center has strict multidisciplinary follow-up (nutritionist, psychologist, endocrinologist, and bariatric surgeon) to ensure patient success.

Another reported case associated with NAGB complications was in the USA in 2015 by Lederer and Whinney [13]. They presented the case of a 20-year-old patient who had undergone this procedure two years prior, thinking it was an adjustable gastric band. In this case, only the band was removed due to significant erosion. In our case, although significant erosion was present, the GS procedure was performed without complications, indicating that undergoing another procedure after band removal is not necessarily contraindicated.

The most recent case published by Duro et al. was in Argentina in 2020 [14]. They described the case of a 64-year-old patient with reflux complications and weight regain. Band removal and conversion to RYGB were performed. They recommend conversion, especially in patients with band-related adhesions. In our case, the presence of adhesions was not a contraindication for performing the GS procedure.

Although there are few cases, and the literature mostly describes RYGB as a treatment option, currently, conversion to GS seems to be a good treatment option with long-term follow-up by a multidisciplinary team. With the increase in bariatric procedures worldwide and in our region, along with the popularization of medical tourism [15], it is important for bariatric surgeons to be aware of these early procedures and to have the skill and knowledge to offer appropriate solutions for patients.

To the best of our knowledge, this is the first case reported of NAGB removal and GS conversion in our country treated successfully.

## Conclusions

The NAGB was a very popular procedure years ago; however, it is now obsolete. Nonetheless, current bariatric surgeons must be familiar with the complications of early bariatric procedures and be capable of making appropriate decisions for patients. Conversion to a GS is a viable treatment option with proper follow-up. More studies and cases are needed to fully evaluate the effectiveness of this surgical option.
